# Disparities in mortality among acute myeloid leukemia‐related hospitalizations

**DOI:** 10.1002/cam4.5084

**Published:** 2022-08-04

**Authors:** Sara Taveras Alam, Deepa Dongarwar, Elyse Lopez, Sarvari Yellapragada, Gustavo Rivero, Quillan Huang, Courtney Miler‐Chism, Martha Mims, Hamisu M. Salihu

**Affiliations:** ^1^ Department of Medicine, Section of Hematology and Oncology Baylor College of Medicine Houston Texas USA; ^2^ Baylor College of Medicine Center of Excellence in Health Equity, Training and Research Houston Texas USA; ^3^ Michael E. DeBakey VA Medical Center, Hematology and Oncology Houston Texas USA; ^4^ Department of Medicine Baylor College of Medicine Houston Texas USA; ^5^ Dan L Duncan Cancer Center Houston Texas USA; ^6^ Department of Family and Community Medicine Baylor College of Medicine Houston Texas USA

**Keywords:** acute myeloid leukemia, HCUP‐NIS, heath disparities, in‐hospital death

## Abstract

Racial and socioeconomic disparities have become apparent in acute myeloid leukemia (AML) outcomes. We conducted a retrospective cohort study of hospitalizations for adults with a diagnosis of AML from 2009 to 2018 in the Nationwide Inpatient Sample (NIS). We categorized patients' ages in groups of <60 years and ≥60 years and stratified them by reported race/ethnicity. Exposures of interest were patient sociodemographics, hospital characteristics, and Elixhauser‐comorbidity Index. Outcome of interest was in‐hospital death. Statistical analyses included survey logistic regression to generate adjusted odds ratios (aORs) and 95% confidence intervals (CIs) to quantify the independent associations between patient characteristics and mortality. Of 622,417 AML‐related hospitalizations, 57.6% were in patients ≥60 years. The overall rate of in‐hospital death was 9.4%. Compared to patients <60, older patients experienced a higher rate of in‐hospital death. In both age groups and in all ethnicities, mortality decreased over time. Differences in mortality were observed based on gender, payer, hospital location, and teaching status. For hospitalizations in patients ≥60, NH‐Black race was associated with inferior in‐hospital death outcomes (OR 1.17; CI 1.08–1.28). Urban teaching hospitals were associated with a 38% increase (OR 1.38; CI 1.06–1.80) in inpatient mortality in patients <60 and a 15% decrease (OR 0.85; CI 0.77–0.95) in inpatient mortality in patients ≥60. Our results highlight the increased need to recognize the role of race/ethnicity and socioeconomic factors and their contribution to disparate outcomes in AML.

## Graphical abstract

We evaluated sociodemographic disparities in the mortality of hospitalized patients with acute myeloid leukemia (AML) through a retrospective cohort study of hospitalizations for adults with a diagnosis of AML from 2009 to 2018 in the United States using the Nationwide Inpatient Sample (NIS). Race, gender, and teaching hospital status were found to be associated with disparate outcomes, in addition to established differences in mortality based on age. Our results highlight the increased need to recognize the role of race/ethnicity and socioeconomic factors and their contribution to outcomes in AML.

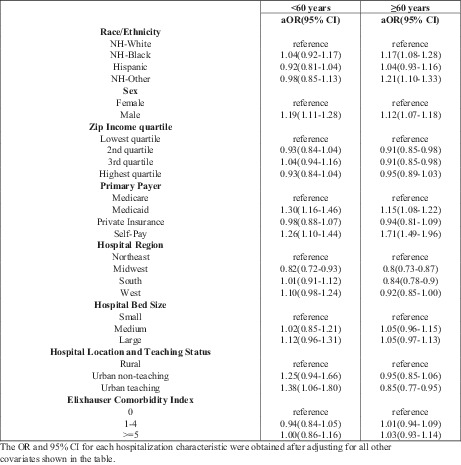

## 1 INTRODUCTION

Acute myeloid leukemia (AML) has been previously reported to have a 5‐year relative survival rate of less than 30%, despite therapeutic advances over the last decade.^1^, ^2^ AML outcomes are traditionally known to be dependent on patient age, performance status, and genetic risk stratification.^3^, ^4^, ^5^ However, racial and socioeconomic disparities have become apparent.^6^, ^7^, ^8^, ^9^, ^10^, ^11^, ^12^, ^13^, ^14^

Despite a lower incidence of AML, Non‐Hispanic Blacks (NH‐Black) and Hispanic patients with AML have been reported to have higher mortality rates than Non‐Hispanic White (NH‐White) patients.^7^, ^8^, ^9^, ^10^ An analysis using the SEER Program along with a survival analysis in Alliance patients indicated that self‐reported African American race is an independent poor survival prognosticator in AML revealing that young (<60 years) African American AML patients have a 28% higher risk of death compared to White AML patients (HR 1.28; 95% CI 1.29–1.37).^11^

Previously published studies on AML‐related hospitalizations have revealed disparities in length of stay and charge per day based on age and race; and disparities in mortality based on volume and teaching status of hospitals,^12^, ^13^, ^14^ but had not evaluated if there are racial disparities in the in‐hospital death of adult patients with AML. We presented the preliminary findings of this study as a poster in the Fourth Annual Summer Research Summit on Health Equity Organized by the Center of Excellence in Health Equity, Training and Research, Baylor College of Medicine and presented our subgroup analysis findings of increased mortality of elderly Hispanic AML patients with comorbidities relative to their counterparts in the same age group without comorbidities at the American Society of Hematology Meeting in 2021.^15^, ^16^ We herein expand our evaluations on in‐hospital death among AML‐related hospitalizations based on sociodemographic characteristics, focusing on the effect of age and race.

## 2 METHODS

### 2.1 Study design and data collection

We conducted a retrospective cohort study using the Healthcare Cost and Utilization Project—Nationwide Inpatient Sample (HCUP‐NIS), the largest database of hospitalizations in the United States of America (USA), from January 1, 2009 to December 31, 2018. The NIS contains discharge data for more than 7 million hospitalizations annually and constitutes a 20% stratified sample of all USA non‐federal, non‐rehabilitation, short‐term community hospitals.^17^ For each hospitalization, up to 30 diagnoses and procedures are able to be captured using the International Classification of Diseases, Ninth Edition, Clinical Modification (ICD‐9‐CM) and the International Classification of Diseases, Tenth Edition, Clinical Modification (ICD‐10‐CM) which became available from October 1, 2015. The unit of analysis when using the NIS is the hospitalization, or discharge, as opposed to the individual person since the database does not allow linkage of hospitalizations for the same patient over time due to lack of patient identifiers. This study was exempt by the Baylor College of Medicine Institutional Review Board as NIS data are publicly available and de‐identified.

The study sample consisted of AML‐associated hospitalizations of patients aged 18 years of age and older, identified on the basis of the presence of any diagnosis code indicative of newly diagnosed, in remission, relapsed/refractory acute myeloid leukemia, and myeloid sarcoma (ICD‐9‐CM: 205.0x, 205.3x, 206.0x; ICD‐10‐CM: C92.0x, C92.3x, C92.4x, C92.5x, C92.Ax, C93.0x). We classified patients' ages in groups of <60 years and ≥60 years. Ethnicity was first stratified by reported ethnicity (Hispanic, Non‐Hispanic), and the Non‐Hispanic group was subclassified into White, Black, or other. The primary payer for the hospitalization was divided into Medicare, Medicaid, private, self‐pay, and other (including under/uninsured). As a proxy for socioeconomic status, the Healthcare Cost and Utilization Project provides zip‐code‐level estimates of median household income, grouped into quartiles based on the patient's residence. Hospital factors included census region (Northeast, Midwest, South, West), bed size (small, medium, large), and hospital type (rural, urban‐nonteaching, urban‐teaching). Comorbidities were captured using Elixhauser Comorbidity Index (0, 1–4, 5+).^18^

### 2.2 Statistical analysis

We captured the frequency distribution of patient and hospital characteristics among AML‐related hospitalizations and AML‐related in‐hospital deaths, stratified by patients' age: <60 years and ≥60 years. In each patient and hospital characteristic subgroup, we additionally calculated the incidence of in‐hospital death per 100 AML hospitalizations. We then explored trends of inpatient mortality over time within the study cohort to assess changes in rates of inpatient death over the study period using joinpoint regression.^19^ We used average annual percentage change (AAPC) and 95% confidence intervals to determine the change in trends of outcome during the study period.

Furthermore, we used survey logistic regression to generate adjusted odds ratios (aORs) and 95% confidence intervals (CIs) that measured the independent associations between various patient hospitalization characteristics (exposure) and inpatient death (outcome) among different age groups of patients with AML. Pearson's chi‐squared tests were utilized to examine the bivariate associations between each hospitalization characteristic and inpatient mortality. The covariates for the model were chosen based on the statistically significant associations in the bivariate analyses. The association models were created after removing missing values from all model covariates. A 5% type I error rate was adopted for the calculation of CIs, and appropriate survey weighting was used to generate national prevalence estimates considering the complex sampling design of the NIS.

R (version 3∙6∙1) and RStudio (Version 1∙2∙5001) were utilized for statistical analyses and Joinpoint Regression Program, version 4.7.0.0 (National Cancer Institute) was used for trends analyses. To ensure confidentiality of subjects that fell in a category that contained a sample size less than 10 hospitalizations, we applied cell suppression with a threshold of 10.

## 3 RESULTS

From January 1, 2009 to December 31, 2018, 622,417 AML‐related hospitalizations were identified across the NIS database. Of these hospitalizations, 358,762 (57.6%) were in patients 60 years of age and older. The number of AML‐related in‐hospital deaths was 58,306 (9.4% of all AML hospitalizations), with 42,545 (73%) of these deaths occurring in patients ≥60 years. The baseline sociodemographic characteristics of the patients are compared in Table 1, stratified by patients' age: <60 years and ≥60 years. Most AML‐related hospitalizations were in NH‐White patients (61.6% in <60 years, 74.6% in ≥60 years) and males (50.5% in <60 years and 57.6% in ≥60 years). Hospitalizations in younger patients were primarily covered by private insurance (56.9%), whereas those in older patients were primarily supported by Medicare (71.9%). Most AML‐associated hospitalizations were in large hospitals (73.3% <60 years, 68.8% in ≥60 years) in urban teaching settings (84.5% <60 years, 73.5% in ≥60 years). Almost 90% of hospitalizations were in patients with comorbidities. Compared to younger patients, those ≥60 experienced a higher incidence of in‐hospital death across all sociodemographic characteristics.

**TABLE 1:** Sociodemographic characteristics among AML‐related hospitalizations and those who died in‐hospital with AML, stratified by age groups

	AML		AML in‐hospital death		Rate of in‐hospital death in AML patients	
	*n* = 622,417		*n* = 58,306		*n* = 58,306 (9.4%)	
	<60 years	≥60 years	<60 years	≥60 years	<60 years	≥60 years
	*n* = 263,655 (42.4%)	*n* = 358,762 (57.6%)	*n* = 15,761 (27%)	*n* = 42,545 (73%)		
Race/Ethnicity						
NH‐White	61.6%	74.6%	61.4%	72.4%	6.0%	11.5%
NH‐Black	11.2%	7.3%	11.8%	8.0%	6.3%	13.1%
Hispanic	11.7%	5.4%	11.4%	5.5%	5.8%	12.0%
NH‐Other	8.3%	6.3%	8.3%	7.2%	6.0%	13.7%
Missing	7.2%	6.5%	7.1%	6.9%	5.9%	12.6%
Sex						
Male	50.5%	57.6%	54.8%	60.0%	6.5%	12.4%
Female	49.5%	42.4%	45.2%	40.0%	5.5%	11.2%
Zip income quartile						
Lowest quartile	24.7%	21.3%	25.1%	22.4%	6.1%	12.5%
2nd quartile	25.1%	24.7%	23.8%	23.8%	5.7%	11.5%
3rd quartile	24.6%	25.4%	26.1%	24.5%	6.3%	11.4%
Highest quartile	23.2%	26.8%	22.4%	27.2%	5.8%	12.0%
Missing	2.4%	1.9%	2.6%	2.1%	6.5%	13.5%
Primary payer						
Medicare	11.4%	71.9%	13.7%	73.1%	7.2%	12.1%
Medicaid	23.7%	3.3%	22.3%	2.9%	5.6%	10.5%
Private insurance	56.9%	21.7%	54.4%	19.4%	5.7%	10.6%
Self‐pay	7.8%	3.0%	9.5%	4.4%	7.3%	17.1%
Missing	0.2%	0.2%	0.1%	0.2%	2.7%	15.7%
Hospital region						
Northeast	20.2%	22.0%	20.6%	23.9%	6.1%	12.9%
Midwest	22.9%	23.7%	19.9%	21.9%	5.2%	11.0%
South	36.7%	35.7%	37.7%	34.7%	6.1%	11.5%
West	20.2%	18.6%	21.8%	19.4%	6.4%	12.4%
Hospital bed size						
Small	10.7%	12.3%	9.8%	11.7%	5.5%	11.3%
Medium	15.3%	19.1%	14.3%	19.3%	5.6%	12.0%
Large	73.3%	68.2%	74.9%	68.4%	6.1%	11.9%
Missing	0.7%	0.5%	0.9%	0.6%	7.7%	14.7%
Hospital location and teaching status						
Rural	2.6%	5.2%	2.0%	5.7%	4.6%	13.2%
Urban non‐teaching	12.2%	20.9%	11.6%	22.1%	5.7%	12.6%
Urban teaching	84.5%	73.5%	85.5%	71.6%	6.1%	11.5%
Missing	0.7%	0.5%	0.9%	0.6%	7.7%	14.7%
Elixhauser comorbidity index						
0	10.4%	10.2%	11.0%	10.1%	6.3%	11.8%
1–4	78.9%	78.9%	77.9%	78.8%	5.9%	11.8%
≥5	10.7%	10.9%	11.1%	11.1%	6.2%	12.0%

*Note*: All bivariate associations between each hospitalization characteristic and inpatient mortality yielded a *p*‐value <.05.

**FIGURE 1:**
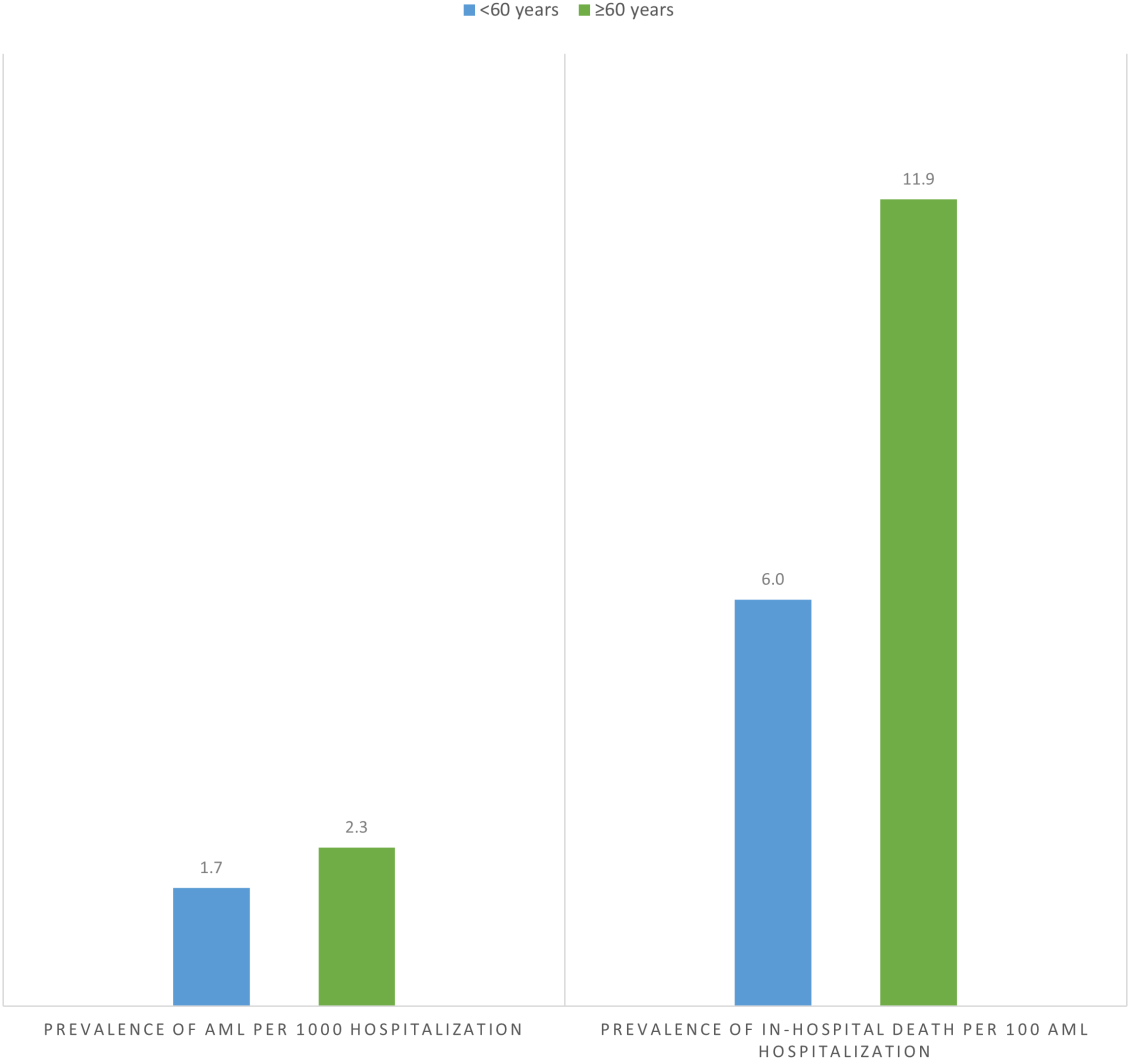
Prevalence of AML hospitalizations and in‐hospital death among AML‐related hospitalizations by patients' age group.

The prevalence of AML hospitalizations was found to be 1.7 per 1000 hospitalizations among patients <60 years and 2.3 per 1000 hospitalizations among patients ≥60 years. The prevalence of in‐hospital death was 6.0 per 100 AML hospitalizations among patients <60 years and 11.9 per 100 AML hospitalizations among patients ≥60 years. Figure 1.

The rate of in‐hospital death decreased over time among AML hospitalizations, however, a persistent gap between the two age groups was apparent with a higher mortality in patients ≥60 years as compared to younger patients. The rate of in‐hospital death in AML patients ≥60 years was 13.6% in 2009 and 10.5% in 2018. The rate of in‐hospital death in AML patients <60 years was 6.8% in 2009 and 5.16% in 2018. The average annual percentage change (AAPC) for in‐hospital death among AML hospitalizations in patients ≥60 years was −2.6% (−3.4%, −1.8%) compared to −3% (−4.9%, −1.2%) in patients <60 years. The AAPC for in‐hospital death among AML hospitalizations across all ages was −2.3% (−3.1%, −1.4%).

**FIGURE 2:**
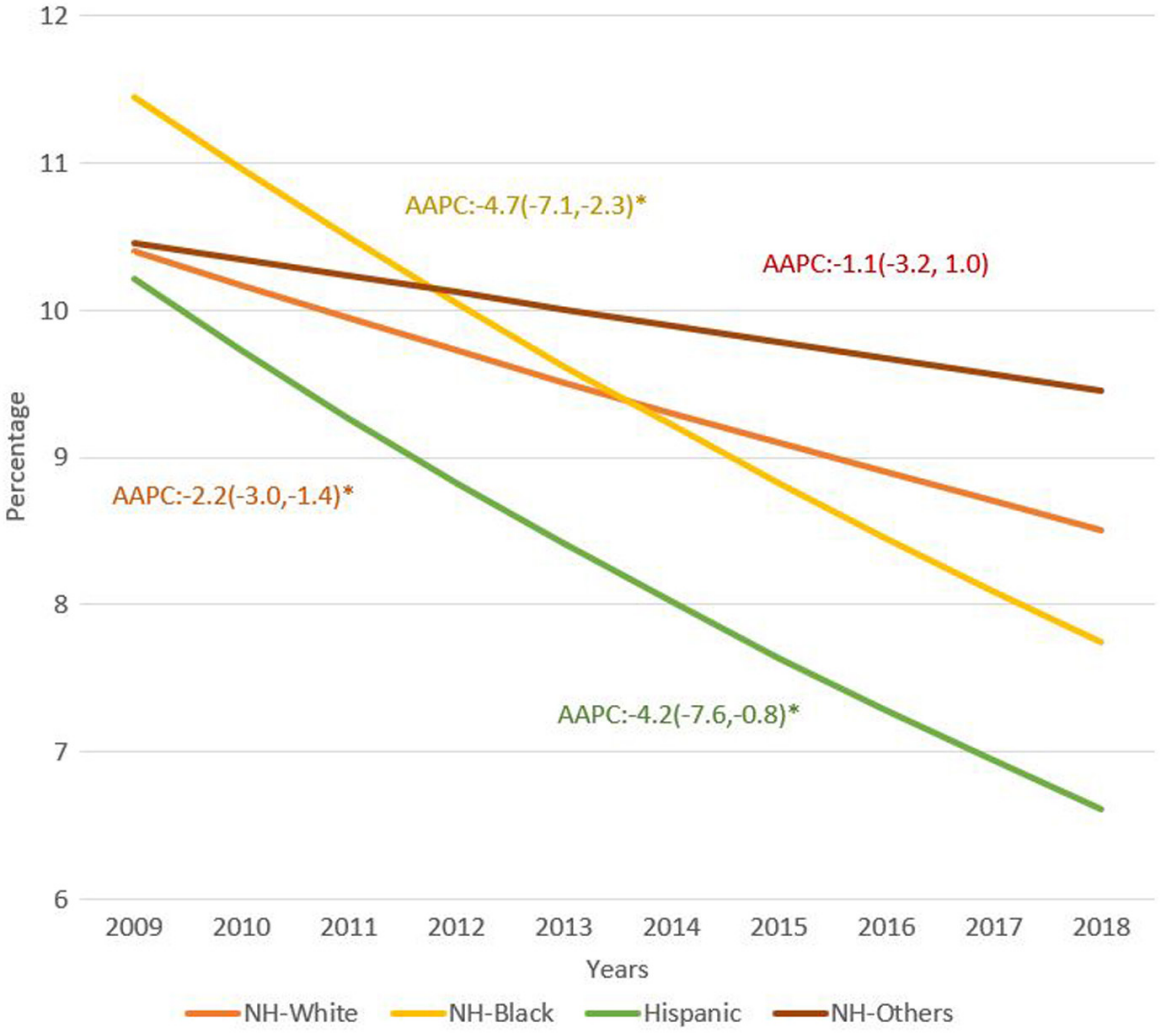
Trends in rates of in‐hospital death among AML‐related hospitalizations by race.

The rate of in‐hospital death decreased over time for AML hospitalizations in all groups, with best outcomes seen in Hispanics. For NH‐White AML patients, the rate of in‐hospital death was 10.46% in 2009 and 8.51% in 2018, with an AAPC for in‐hospital death of −2.2% (−3%, −1.4%). The rate of in‐hospital death in NH‐Black AML patients was 11.45% in 2009 and 7.75% in 2018, with an AAPC of −4.7% (−7.1, −2.3%). The rate of in‐hospital death in Hispanic AML patients was 10.21% in 2009 and 6.61% in 2018% with an AAPC of −4.2% (−7.6, −0.8). The rate of in‐hospital death in NH‐Others was 10.46% in 2009 and 9.45% in 2018% with an AAPC of −1.1% (−3.2, −1.0). Figure 2.

Factors associated with in‐hospital death among AML patients, stratified by age groups are shown in Table 2. There was no difference in mortality based on hospital bed size or comorbidity index.

**TABLE 2:** Factors associated with in‐hospital death among AML patients, stratified by age groups

	<60 years	≥60 years
	aOR(95% CI)	aOR(95% CI)
Race/Ethnicity		
NH‐White	reference	reference
NH‐Black	1.04(0.92–1.17)	1.17(1.08–1.28)
Hispanic	0.92(0.81–1.04)	1.04(0.93–1.16)
NH‐Other	0.98(0.85–1.13)	1.21(1.10–1.33)
Sex		
Female	reference	reference
Male	1.19(1.11–1.28)	1.12(1.07–1.18)
Zip income quartile		
Lowest quartile	reference	reference
2nd quartile	0.93(0.84–1.04)	0.91(0.85–0.98)
3rd quartile	1.04(0.94–1.16)	0.91(0.85–0.98)
Highest quartile	0.93(0.84–1.04)	0.95(0.89–1.03)
Primary payer		
Medicare	reference	reference
Medicaid	1.30(1.16–1.46)	1.15(1.08–1.22)
Private insurance	0.98(0.88–1.07)	0.94(0.81–1.09)
Self‐Pay	1.26(1.10–1.44)	1.71(1.49–1.96)
Hospital region		
Northeast	reference	reference
Midwest	0.82(0.72–0.93)	0.8(0.73–0.87)
South	1.01(0.91–1.12)	0.84(0.78–0.9)
West	1.10(0.98–1.24)	0.92(0.85–1.00)
Hospital bed size		
Small	reference	reference
Medium	1.02(0.85–1.21)	1.05(0.96–1.15)
Large	1.12(0.96–1.31)	1.05(0.97–1.13)
Hospital location and teaching status		
Rural	reference	reference
Urban non‐teaching	1.25(0.94–1.66)	0.95(0.85–1.06)
Urban teaching	1.38(1.06–1.80)	0.85(0.77–0.95)
Elixhauser comorbidity index		
0	reference	reference
1–4	0.94(0.84–1.05)	1.01(0.94–1.09)
≥5	1.00(0.86–1.16)	1.03(0.93–1.14)

*Note*: The OR and 95% CI for each hospitalization characteristic were obtained after adjusting for all other covariates shown in the table.

For patients 60 years of age or older, the risk of in‐hospital death was greater in NH‐Blacks (Odds Ratio [OR] 1.17; 95% Confidence Interval [CI]:1.08–1.28) as compared to NH‐Whites. The risk of in‐hospital death was greater in males (OR 1.19; CI 1.11–1.28 for patients younger than 60 and OR 1.12; CI 1.07–1.18 for ages 60 and older) compared to females.

Across all ages, the risk of in‐hospital mortality was higher among self‐pay hospitalizations (OR 1.26; CI 1.10–1.44 for patients younger than 60 and OR 1.71; CI 1.49–1.96 for ages 60 and older) or hospitalizations covered by Medicaid (OR 1.30; CI 1.16–1.46 for patients younger than 60 and OR 1.15; CI 1.08–1.22 for patients ages 60 and older) compared to hospitalizations covered by Medicare.

The risk of in‐hospital death was lower for hospitals in the Midwest among patients younger than 60 (OR 0.82; CI 0.72–0.93), as compared to hospitals in the Northeast. For patients ≥60 years, the risk of in‐hospital mortality was lower in the Midwest (OR 0.8; CI 0.73–0.87) and South (OR 0.84; CI 0.78–0.9), as compared to hospitals in the Northeast. Urban teaching hospitals were associated with a 38% increase (OR 1.38; CI 1.06–1.80) in inpatient mortality in patients <60 and a 15% decrease (OR 0.85; CI 0.77–0.95) in inpatient mortality in patients ≥60.

## 4 DISCUSSION

We identified more than half a million AML‐related hospitalizations in adults over the span of a decade utilizing the HCUP‐NIS database, making this the largest population study evaluating disparities related to AML outcomes to date. There were 58,300 in‐hospital deaths among these hospitalizations with 73% of deaths in patients 60 years of age and older. AML is known to have a worse outcome with regards to remission rates and overall survival in older patients,^4^ and previous population studies had shown an higher in‐hospital death among patients ≥60.^12^ Our study further explores in‐hospital death among AML‐related hospitalizations based on sociodemographic characteristics, focusing on the effect of age and race/ethnicity.

We found that the rates of in‐hospital death did improve over time, however, we noted a persistent gap between age groups with a higher mortality in AML‐related hospitalizations among patients ≥60. This is consistent with reported relative survival rate trends across all races on SEER, stratified by age.^1^ For patients 60 years of age or older, the risk of in‐hospital death was 17% greater in self‐reported NH‐Blacks as compared to NH‐Whites, however, there was no statistically significant difference observed across races in patients younger than 60. This finding contrasts with a previous report that young (<60 years) African American AML patients have a 28% higher risk of death compared to White AML patients.^11^ Since the NIS data are based on AML‐related hospitalizations and our outcome was in‐hospital death, we acknowledge that our results were limited by not accounting for out of hospital deaths, repeated hospitalizations for the same patient, cytogenetic risk, performance status, or treatment received including transplant status since this information was not available for our study population.

Mortality improved over time across all races/ethnicities and we noted a more notable improvement among Hispanics and NH‐Blacks when compared to NH‐Whites and Others leading to a decreased disparity. In‐hospital death among AML‐related hospitalizations was lower in Hispanics and NH‐Blacks than among NH‐Whites in 2018. The same limitations described above apply and the differences in these trends over time warrant further evaluation.

Male sex was associated with an elevated risk of in‐hospital death across all age groups. An increased incidence of AML in males and increased death rates in males across all ethnicities were reported previously^1^ but remains unexplained.

Self‐pay hospitalizations were associated with an increased risk of in‐hospital mortality across all ages. Uninsured patients with AML have been previously found to have worse outcomes and are more likely to remain untreated than insured patients, presumably due to hesitancy to seek care and cost of care.^20^

To our surprise, the association between urban teaching hospitals and inpatient mortality among AML‐related hospitalizations was age‐dependent, with increased inpatient mortality in <60 and decreased inpatient mortality in patients ≥60. High‐volume centers and academic centers have been associated with improved outcomes in patients with AML.^13^, ^21^ The infrastructure at such centers may allow the support of AML treatment in an outpatient setting more so than non‐academic centers.^22^ Our findings of increased inpatient mortality in younger patients at academic centers might be because younger patients were prone to be treated more aggressively, and were also less likely to accept hospice care. The decreased inpatient mortality at academic centers for older patients may be explained by enhanced center expertise, access to clinical trials, infrastructure that allows more outpatient care and older patients with AML potentially having a higher likelihood of death at home if such patients should accept hospice. These are speculations that necessitate further investigation.

Our study highlights that there are many nuances to disparate outcomes in AML that require further investigation and correlation with studies where overall survival outcomes are measured and sociodemographic characteristics are available for review along with the presence of comorbidities, cytogenetic risk, and treatment received. Such a step is important to uncover, at a more granular level, pathways that could explain the heightened risks of in‐hospital mortality in certain sociodemographic subpopulations as reported herein.

## AUTHOR CONTRIBUTION

STA and DD designed research. DD analyzed data. STA, DD, HS, and EL wrote the paper. SY, GR, MM, CMC, and QH reviewed paper.

## FUNDING INFORMATION

Health Resources and Services Administration (HRSA) Grant number 1 D34HP31024–01‐00 for *Baylor College of Medicine* *(BCM) Center of Excellence in Health Equity, Training & Research* supported this research.

## CONFLICT OF INTEREST

No COI declared.

## ETHICS STATEMENT

This study was exempt by the Baylor College of Medicine Institutional Review Board as NIS data are publicly available and de‐identified.

## Supporting information


Figure S1

## Data Availability

The data supporting this study are available in the Healthcare Cost and Utilization Project's Nationwide Inpatient Sample (HCUP‐NIS), at https://www.hcup‐us.ahrq.gov/tools_software.jsp
