# Supplementary figures and images for: Disparities in mortality among acute myeloid leukemia‐related hospitalizations
Source: Cancer Med. 2022 Aug 4;12(3):3387–94. doi: 10.1002/cam4.5084 (PMC9939120; doi:10.1002/cam4.5084)

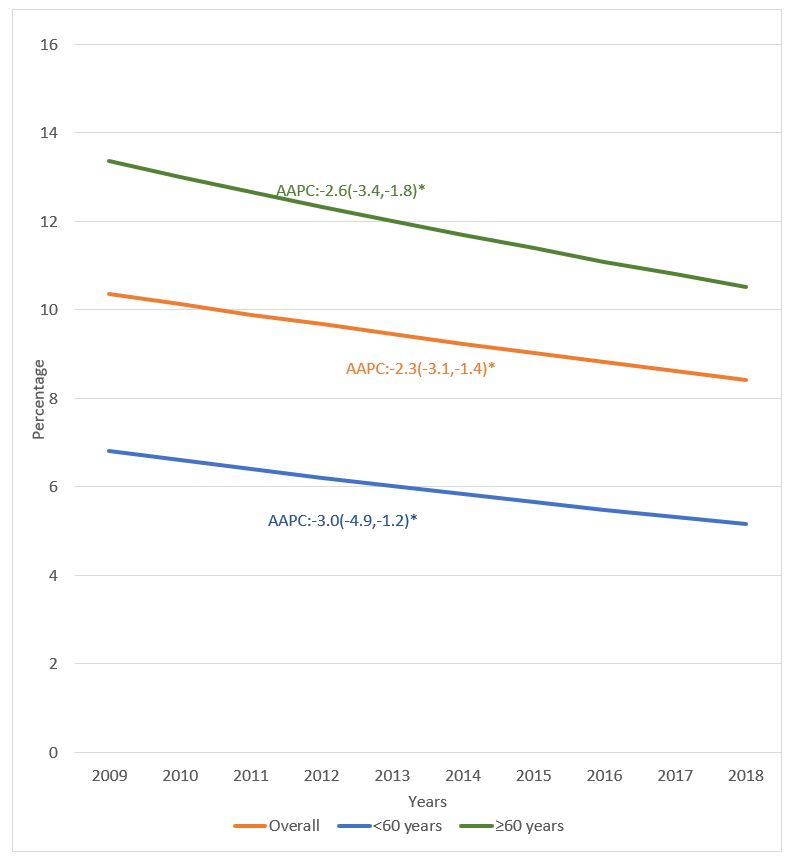

Supplement: Supplementary file 1 — Figure S1 [file CAM4-12-3387-s001.JPG]
